# Probiotics and enzymes in weaned piglets’ diets can lower belly nosing frequency and improve performance and nitrogen balance

**DOI:** 10.1007/s11250-026-04893-2

**Published:** 2026-02-26

**Authors:** Bruno Braga Carnino, Ana Ligia Braga Mezzina, Nadia de Almeida Ciriaco Gomes, Guilherme Cunha Gottschall, Bruno Bracco Donatelli Muro, Leticia Gomes de Morais Amaral, Cesar Augusto Pospissil Garbossa, Vinícius de Souza Cantarelli

**Affiliations:** 1 Swine Research Laboratory, Animal Nutrition and Production Department, School of Veterinary Medicine and Animal Science, University of São Paulo, Pirassununga, SP Brazil; 2 Animal Science Department, Federal University of Lavras, Lavras, MG Brazil

**Keywords:** Nutrition, Swine, Welfare, Metabolism, Nursery, Antibiotics

## Abstract

Two studies were conducted to evaluate the effect of two classes of feed additives, probiotics and enzymes, on performance, diarrhea incidence, belly nosing frequency, and nutrient metabolism in post weaned piglets. The hypothesis was that the association of probiotics and multienzymes would reduce diarrhea, improve behavioral outcomes, and enhance nutrient digestion and absorption, leading to better performance and health. In the first trial, 80 weaned piglets, barrows and gilts, were distributed in four treatments using a randomized block design based on the initial weight. Each treatment had five replicates (pens), housing four piglets. The treatments were as follows: CON – no feed additives; AB – antibiotics (lincomycin and colistin); FA - probiotic (*Bacillus subtilis*, 3 × 10^8^ CFU/g) and multienzymes (exoglycanase, endoglycanase, protease, pectinase, polygalacturonidase, and β-amylase); AB + FA - antibiotic, probiotics, and multienzymes. Piglets were weighed at the start of the trial and on days 7, 14, 21, 28, and 35. Feed intake was recorded to calculate body weight (BW), average daily gain (ADG), average daily feed intake (ADFI), and the feed-to-gain ratio (F: G). Diarrhea score and belly nosing behavior were evaluated daily by the same observer. In the second trial, 20 barrows were selected at the end of the nursery phase, allocated in a randomized block design based on their initial weight, and housed in metabolic cages. For 12 days, the piglets were subjected to the same four treatments as in the first trial. Total feces and urine were collected during the last four days to assess the dry matter digestibility coefficient, gross energy digestibility coefficient, digestible energy, metabolizable energy, absorbed nitrogen (NABS), retained nitrogen, and the ratio of retained to absorbed nitrogen. Statistical analysis was performed in SAS, linear mixed models and Tukey were used for performance variables and digestibility. For fecal score analysis and belly nosing frequency, Kruskal-Wallis and Dunn’s test were adopted. Means were considered statistically different when *p* < 0.05. In the first study, the BW and ADG of the AB + FA group were 7.56% and 56.86% higher, respectively, than those of the AB group. During the first week, the AB group had a lower ADFI compared to all other treatments. The AB + FA group exhibited a 20.17% lower frequency of belly nosing compared to piglets in the CON group. Additionally, the FA group had 7.14% higher NABS compared to the CON group. Within the conditions of the present study, supplementing piglet diets containing antibiotics with probiotics and a multienzyme blend appeared to improve performance, reduce undesirable behaviors, and enhance protein utilization.

## Introduction

Early weaning is commonly practiced in commercial pig production to maximize productivity; however, this strategy can be stressful for piglets and cause negative side effects (Van Kerschaver et al. 2023). Post-weaning, piglets’ feed intake lowers, which disrupts intestinal normal function and increased the risk of dysbiosis and post-weaning diarrhea (Tang et al. 2022). Antibiotics in feed were introduced to mitigate the negative effects of weaning and to enhance the performance and intestinal health of piglets during this critical period. Although this strategy has proven effective, the risk of cross-resistance, where bacteria resistant to one class of antibiotics may develop resistance to others, has led to the ban of antibiotics in animal feed in regions such as Europe (Diana et al. 2017).

Alternative feed additives, such as probiotics and enzymes have emerged as promising strategies to mitigate the challenges of weaning. Probiotics modulate gastrointestinal microbiota by competitively inhibiting other bacteria growth and favoring non-pathogenic microorganisms, which stimulate the intestinal immune system (Duarte et al. 2020; Mekonnen et al. 2020). Enzymes, in turn, facilitate nutrient breakdown, enhancing digestion efficiency and reducing the stress associated with nutritional transitions (Boontiam et al. 2022; Duarte et al. 2020). By increasing nutrient utilization, enzymes reduce the amount of substrate available for microbial fermentation in the large intestine and decrease inflammatory stimuli; proteins for instance, can induce dysbiosis and diarrhea when fermented (Pieper et al. 2016). Although feed additives can replicate some of the health-promoting effects of antibiotics, they are often incapable of fully replacing antibiotics in commercial farms when only one category is added.

For this reason, associating feed additives has become common practice in pig and livestock nutrition, with some combinations even having specific names such as symbiotics (the combination of probiotics and prebiotics) (Martyniak et al. 2021). The combination of probiotics and enzymes is interesting as they do not share a common mechanism of action, thus their effects are not compromised by the presence of the other. Enzymatic breakdown increases the carbohydrate-to-protein ratio in the intestinal substrate, promoting the production of key microbial metabolites such as short-chain fatty acids (Bedford and Apajalahti 2022; Liao and Nyachoti 2017).

Adding enzymes that can target non-starch polysaccharides, such as pectinase, polygalacturonidase, xylanase and glucanases, which depolymerize these complex carbohydrates, releasing fermentable oligosaccharides and reducing chyme viscosity (Choi et al. 2024; Drochner et al. 2004; Kiarie et al. 2013). The carbohydrates liberated can be used by probiotics such as *Bacillus subtilis* to proliferate (Mingmongkolchai and Panbangred 2018) and promote their benefits. *B. subtilis* is a spore forming, temperature resistant bacteria commonly used in pig nutrition due to its capacity to proliferate in the intestines and release antibiotic substances (Tam et al. 2006; Stein 2005). The combined use of *B. subtilis* and xylanase has already been reported to improve average daily gain and body weight in piglets (Duarte et al. 2020). *B. subtilis* also has been reported to produce enzymes (Latorre et al. 2016), including amylases and proteases, which could also help improve the action of the exogenous enzymes. In broilers, enzymes and a lactobacillus based probiotic improved expression of tight junction proteins and altered the intestinal microbiota which resulted in higher propionate production (Gao et al. 2022). Both feed additives help lower overall intestinal inflammation by different mechanisms, i.e. probiotics by competitive inhibition and enzymes by fermentable substrate control.

Therefore, combining these additives in the nutrition of weaned piglets may be interesting to reduce negative impacts of early weaning by improving immune status and nutrient digestion. Given these considerations, this study aims to evaluate alternatives to antibiotic use by assessing the performance, behavior, digestibility, and intestinal health of weaned pigs supplemented with three different additives and their combinations: antibiotics alone, probiotics with multienzymes, and a combination of all three.

## Materials and methods

### Health and performance trial

#### Animals, housing, and diets

A total of eighty weaned piglets (barrows and gilts) of Topigs^®^ lineage were weaned at 20 days of age and average weight of 4.68 ± 0.41 kg and housed in nursery facilities. To minimize initial microbiota differences the animals used in this study originated from the same farrowing batch and were weaned and housed on the same day. Transportation was standardized for all piglets. Prior to housing, nursery facilities underwent cleaning and disinfection procedures with a broad-spectrum disinfectant, followed by a five-day sanitary downtime. Facilities temperature was monitored using strategically placed thermometers and controlled through manual regulation of side curtains. All management practices were conducted in accordance with the thermal and environmental requirements appropriate for the piglet’s age group.

Pens were equipped with slatted floor stalls, suspended at 1.20 m in height and dimensions of 2.00 × 1.20 m, semi-automatic feeders, and nipple drinkers. During experimental procedures, animals received water and feed *ad libitum*. Piglets received isonutritive and isocaloric diets that met all nutrient requirements for each phase following Rostagno et al. (2005) – Table 1. These guidelines were chosen to provide precise nutrient formulations tailored to the specific needs of pigs in Brazilian production systems and to support optimal growth performance under tropical climate environments.

**Table 1:** Percentage and bromatological composition of basal diets in the pre-initial I, pre-initial II and initial phases

Ingredients, %	Experimental Diets ^‡^		
	Pre initial I	Pre initial II	Initial
Corn	14.70	45.23	53.57
Gelatinazed corn	15.00	0.00	0.00
Soybean meal 46%	25.25	29.00	27.40
Bakery by products	20.00	6.00	10.80
Dextrose	5.00	2.00	0.00
Dairy concentrate	5.09	0.00	0.00
Energetic concentrate	6.00	8.00	0.00
Soy oil	0.00	0.40	1.00
NaCl	0.35	0.35	0.50
Limestone	0.00	0.07	1.00
Dicalcium phosphate	1.61	1.52	0.92
Sugar	0.00	2.00	3.00
Inert material	1.00	1.00	1.00
Soybean protein isolate	3.93	3.25	0.00
Sodium bicarbonate	0.20	0.20	0.00
Senamix	0.10	0.10	0.06
Mineral and vitamin premix^†^	0.20	0.20	0.25
Colina 60%	0.01	0.00	0.04
L-Lysine	0.60	0.40	0.28
Threonine	0.23	0.07	0.08
L-Valine	0.20	0.02	0.00
L-Tryptophan	0.03	0.00	0.00
MHA Novus	0.31	0.11	0.09
Tecnaroma ZTA Note	0.12	0.03	0.00
Tecnaroma Milk Swe	0.04	0.02	0.00
Power Sweet	0.03	0.03	0.00
Banox	0.00	0.00	0.005
Nutrient calculated composition			
Crude Protein (%)	19.98	20.11	17.94
Metabolizable energy (kcal/kg)	3552.87	3350.00	3319.66
Lactose (%)	12.00	8.00	0.00
SID Lysine (%)	1.45	1.34	1.04
SID Methionine + Cysteine (%)	0.82	0.67	0.58
SID Threonine (%)	0.89	0.76	0.67
SID Tryptophane (%)	0.25	0.23	0.19
Calcium (%)	0.85	0.70	0.74
Total phosphorus (%)	0.66	0.65	0.49

^†^Mineral and Vitamin premix contets per kg: Vitamin A, 40.000 UI; vitamin D_3_, 6.000 UI; vitamin E, 250 UI; vitamin K, 10 mg; vitamin B_1_, 5 mg; vitamin B_2_, 18 mg; vitamin B_6_, 6 mg; vitamin B_12_, 120mcg; Folic acid, 1,5 mg; Biotin, 100mcg; Niacin, 150 mg; Pantothenic acid, 63 mg; Fumaric acid 15.000 mg; Choline, 1600 mg; antioxidant, 660 mg; flavoring, 2500 mg; Cb, 1,6 mg; Cu, 1000 mg; Fe, 500 mg; I, 1,6 mg; Mg, 230 mg; Se, 0,8 mg; Zn, 450 mg

^‡^Diets description: pre-initial I – from to 7 kg of BW; pre-initial II – from 7 to 15 kg of BW; initial – from 15 to 30 kg of BW

#### Experimental design

Piglets were distributed using a complete randomized block design, with initial body weight and sex as blocking factors. Low, medium, and high body weights were balanced across pens and evenly distributed among treatments to ensure uniformity. Each pen, housing four piglets (two barrows and two gilts), served as the experimental unit, and average pen performance was used for analysis. Pens were randomly assigned to one of four treatments, with five replicates per treatment. Treatments were as follows: CON - basal diet formulated to meet the nutritional requirements of each phase, without antibiotics, probiotics or enzymes; ATB - basal diet with inclusion of antibiotic, specifically lincomycin and colistin; FA - basal diet with inclusion of the probiotic *Bacillus subtilis* (3 × 10^8^ CFU/g of product) and a multienzyme blend of exoglucanase, endoglucanase, protease, pectinase, polygalacturonidase, and β-amylase (BioAmin – FATEC/Trouw Nutrition^®^); AB + FA - basal diet with inclusion of antibiotic, probiotic and multienzyme blend. All diets included zinc oxide at 1500 mg/kg throughout the entire trial. Experimental activities were carried out over 35 days, divided into three phases, based on the dietary changes: pre-initial 1 (days 0 to 14), pre-initial 2 (days 15 to 28), and initial (days 29 to 35).

The inclusion ratio of probiotic and multienzyme blend was 1.5% during the pre-initial 1 and − 2, and 2% in initial phase. The decision to include these additives was based on their documented potential to promote growth and health of piglets, as described in the literature. Probiotics support the proliferation of beneficial bacteria, strengthen immunity, and reduce post-weaning diarrhea. Multienzyme blends enhance nutrient digestibility, improving absorption and reducing environmental waste. When used together, probiotics and enzymes exhibit a complementary effect, supporting gut health and nutrient utilization (Duarte et al. 2020; Mekonnen et al. 2020; Pieper et al. 2016).

#### Data collection

To lower the influence of stress in the observed treatment response, after the beginning of the trial no redistribution of piglets between pens was performed, nor were any new piglets introduced into the experimental group. Animal procedures were kept at a minimum necessary for data collection. Data collection was conducted by a single observer, who maintained control over movement within the facilities, thereby ensuring standardization and minimizing external interference with the collected data.

Piglets were individually weighed at the start, and on days 7, 14, 21, 28, and 35 to determine body weight (BW) and average daily gain (ADG). The feed offered and leftovers were measured daily to calculate the average daily feed intake (ADFI) and feed-to-gain ratio (F: G). Feces were scored daily by the same observer, following De Cupere et al. (1992) methodology. Scores ranged from 0 to 3, as follows: score 0 - normal stools, firm and dry feces; score 1 - pasty feces; score 2 - thick and fluid feces and score 3 - watery feces. Fecal score was calculated as the sum of the fecal scores over a given period, divided by the number of days. Diarrhea was defined as a fecal score of 3 sustained for at least two consecutive days. Belly nosing behavior was observed daily from 7:00 a.m. to 8:00 a.m. and from 4:00 p.m. to 5:00 p.m. Each belly nosing event was recorded and attributed to an individual piglet. By the end of the trial, the frequency of belly nosing events and total events were analyzed.

### Digestibility trial

#### Animals, housing, diets, and experimental design

Fifty-five days after the start of the health and performance trial, twenty barrows with an average weight of 15 kg were selected from the initial 40. The selected barrows were housed in metabolic cages within controlled temperature room. The initial diet (Table 1) was provided based on the metabolic weight (BW^0.75^) and water was offered *ad libitum*. The experimental design and treatments were identical to those used in the health and performance trial, however in this trial, the experimental unit was the individual barrow.

#### Data collection

The digestibility trial lasted for twelve days, with the first eight days serving as an adaptation period and the final four days dedicated to fecal and urine collection. This schedule allowed the animals to acclimate to the experimental conditions before sample collection. During the adaptation period, the animals were acclimatized to the metabolic cages and voluntary feed intake was adjusted. Feces and urine collection were performed according to Sakomura and Rostagno (2007) methodology.

Chromic oxide (Cr_2_O_3_) at 0.2% was used as a digestibility indicator. During the collection period, feces were collected daily, stored in plastic bags and kept in a freezer at -10 °C until processing. Prior to analysis, samples were defrosted, weighed, dried in a forced-air oven at 55–65 °C, finely ground using a 1 mm sieve for homogeneity, and stored in airtight containers. Urine was collected separately from feces, in a plastic bucket covered by a filter and containing 20 ml of hydrochloric acid (HCl) diluted in 1:1 ratio with distilled water, to maintain a pH < 3 and prevent contamination and nitrogen losses. All procedures followed Sakomura and Rostagno (2007) methodology.

Nitrogen content in the fecal and urine samples was determined using the Kjeldahl method, which quantifies total nitrogen excretion to calculate nitrogen absorption (NABS) and retention (NRET). Gross energy (GE) content was determined through bomb calorimetry, allowing the calculation of digestible energy (DE) by subtracting fecal energy loss from total intake. Urinary energy was also analyzed using bomb calorimetry to measure energy lost through excreted compounds.

The analyzed variables included dry matter digestibility coefficient (DMDC), gross energy digestibility coefficient (GEDC), digestible energy (DE), metabolizable energy (ME), absorbed nitrogen (NABS), retained nitrogen (NRET) and the ratio between retained by absorbed nitrogen (NRETABS). The DMDC and GEDC were determined by calculating the difference between intake and fecal excretion of dry matter and gross energy, respectively, expressed as a percentage of intake. DE was obtained by subtracting fecal energy loss from gross energy intake, while ME was determined by further subtracting urinary and gaseous energy losses from DE. NABS was calculated as the difference between nitrogen intake and fecal nitrogen excretion, while NRET was obtained by subtracting urinary nitrogen excretion from NABS. Finally, NRETABS was expressed as the ratio of NRET to NABS, indicating the efficiency of nitrogen utilization. These calculations were performed based on standard digestibility and metabolism balance equations, ensuring accurate assessment of nutrient and energy utilization, described by Sakomura and Rostagno (2007). All analyses were performed at the Animal Research Laboratory of the Federal University of Lavras.

### Statistical analysis

Statistical analyses of performance variables were conducted using linear mixed models, with the pen as the experimental unit and block as a random effect. Analyses were executed in SAS 9.4. Model residuals were tested for normality using the Shapiro-Wilk test (UNIVARIATE procedure) and visually assessed through histograms and Q-Q plots. Homoscedasticity was evaluated with the Breusch-Pagan test (HETERO option in PROC REG) and residuals versus fitted value plots. Data violating normality or homoscedasticity assumptions were transformed accordingly. Non-normal data were addressed using the PROC RANK procedure in SAS with the NORMAL option. For parametric performance and digestibility variables, post-hoc comparisons were performed using Tukey’s test, and results were presented as least square means (LSMEANS) with the standard error of the mean (SEM). Nonparametric variables such asdiarrhea incidence and belly-nosing frequency were analyzed using the Kruskal-Wallis test, with Dunn’s test for pairwise comparisons. A significance level of 5% (*p* < 0.05) was determined for all tests. When statistical significance was observed in parametric variables, effect size was calculated using Cohen’s d to assess the magnitude of treatment effects. The interpretation followed Cohen’s conventional thresholds: small (0.2), medium (0.5), and large (≥ 0.8).

## Results

### Performance and health trial

In the performance and health trial, the piglets fed with AB + FA had BW 7.56% higher (*p* = 0.039), ADG 56.86% higher (*p* = 0.025) and ADFI 39.81% higher than the piglets receiving only antibiotics during the first seven days – Table 2. In this same period, FA and CON also had higher ADFI, 38.83% and 41.75% higher, respectively, than AB group.

At 28 days, AB + FA group outperformed both AB and CON groups. AB + FA had 12.17% higher BW (*p* = 0.020) and 18.50% higher ADG (*p* = 0.017) than AB, while also having BW 10.79% and ADG 15.95% higher than CON. This difference continued at 35 days, where AB + FA had BW 11.69% (*p* = 0.050) and ADG 15.87% (*p* = 0.050) higher than AB. Besides, AB + FA piglets had BW and ADG, 13.33% and 17.11% respectively, higher than piglets on the control group.

**Table 2:** Body weight (BW), average daily weight gain (ADG), average daily feed intake (ADFI) and feed ratio-to-gain (F: G) of piglets in nursery phase consuming diets with different additives†

Item	CON	AB	FA	AB + FA	SEM^*^	Cohen’s D	*P*-value
Initial weight (kg)	4.68	4.71	4.68	4.71	0.062	-	0.818
1 to 7 days							
BW (kg)	5.67^ab^	5.42^b^	5.62^ab^	5.83^a^	0.192	0.373	0.039
ADG (kg/day)	0.141^ab^	0.102^b^	0.134^ab^	0.160^a^	0.026	0.327	0.025
ADFI (kg/day)	0.146^a^	0.103^b^	0.143^a^	0.144^a^	0.023	-0.039	0.033
F: G	1.11	1.26	1.32	0.96	0.349		0.408
1 to 14 days							
BW (kg)	7.80	7.50	7.59	8.10	0.450	-	0.204
ADG (kg/day)	0.223	0.200	0.207	0.243	0.030	-	0.164
ADFI (kg/day)	0.242	0.213	0.234	0.254	0.029	-	0.217
F: G	1.10	1.10	1.22	1.07	0.136	-	0.371
1 to 21 days							
BW (kg)	10.46	10.45	10.54	11.27	0.728	-	0.273
ADG (kg/day)	0.276	0.274	0.279	0.313	0.033	-	0.257
ADFI (kg/day)	0.363	0.333	0.357	0.371	0.035	-	0.379
F: G	1.34	1.24	1.32	1.2	0.115	-	0.214
1 to 28 days							
BW (kg)	13.81^b^	13.64^b^	14.06^ab^	15.30^a^	0.768	0.868	0.020
ADG (kg/day)	0.326^b^	0.319^b^	0.335^ab^	0.378^a^	0.026	0.894	0.017
ADFI (kg/day)	0.468	0.435	0.459	0.480	0.031	-	0.195
F: G	1.46	1.38	1.42	1.28	0.126	-	0.183
1 to 35 days							
BW (kg)	17.70^b^	17.96^b^	18.64^ab^	20.06^a^	1.296	0.814	0.050
ADG (kg/day)	0.374^b^	0.378^b^	0.400^ab^	0.438^a^	0.036	0.795	0.050
ADFI (kg/day)	0.703	0.665	0.710	0.734	0.054	-	0.282
F: G	1.92	1.77	1.81	1.70	0.167	-	0.240

Means on the same line followed by different superscript lowercase letters were significantly different in the Tukey test (*P* < 0.05)

*SEM = standard error of the mean

Effect sizes (Cohen’s d) were calculated for the pairwise comparison between the CON and AB + FA groups, reflecting the lowest and highest treatment responses, respectively

†CON: basal diet formulated to meet the nutritional requirements of each phase. without the inclusion of antibiotic; ATB: basal diet with inclusion of antibiotic, specifically, lincomycin and colistin; FA: basal diet with inclusion of probiotic and multienzyme blend, the probiotic was Bacillus subtilis (3 × 108 CFU/g of product) and a multienzyme blend of exoglycanase, endoglycanase, protease, pectinase, polygalacturonidase, and β-amylase (BioAmin – FATEC/Trouw Nutrition^®^); AB + FA: basal diet with inclusion of antibiotic, probiotic and multienzyme blend

**Fig. 1:**
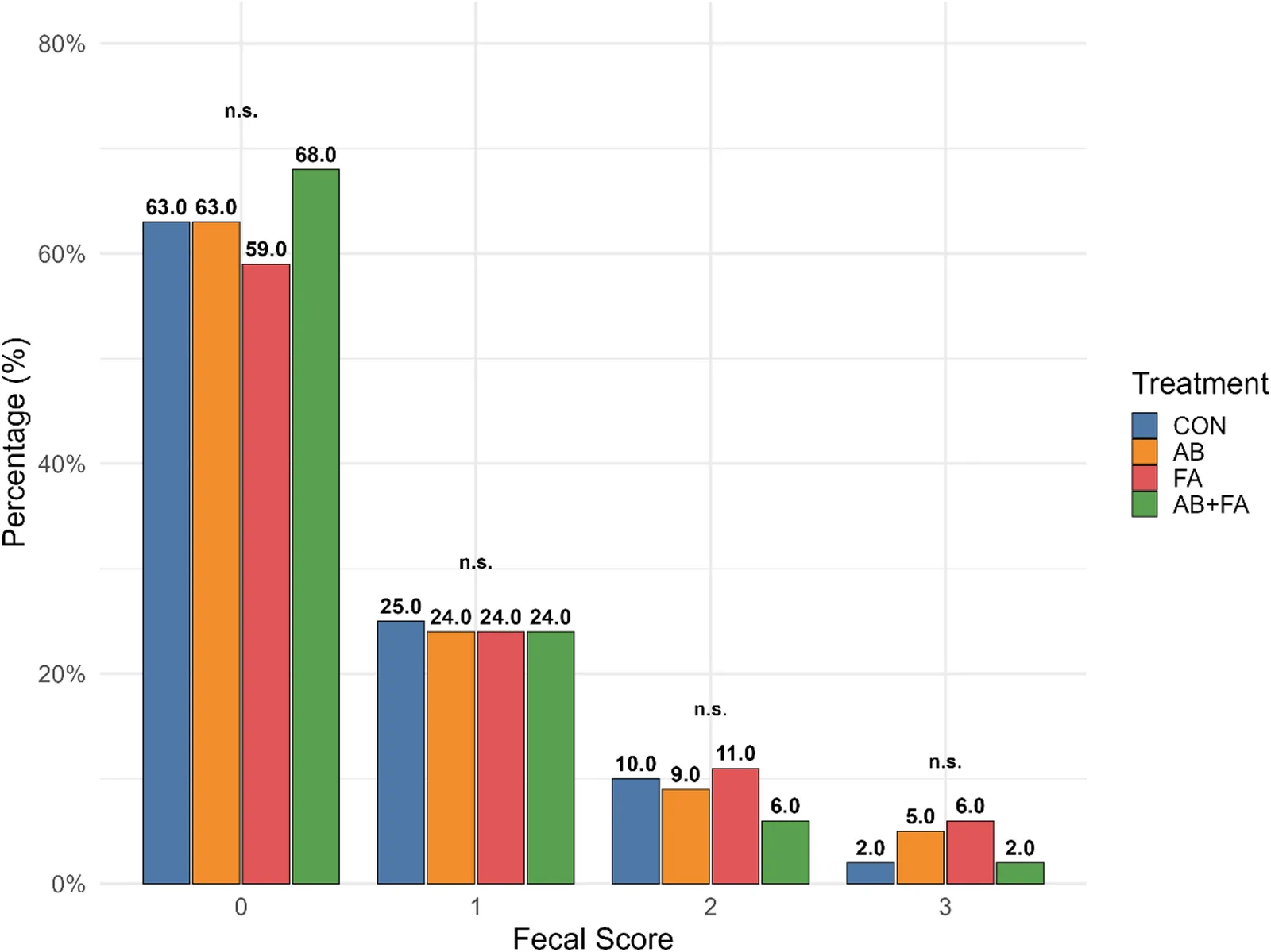
Daily fecal score of pigs in the initial stage receiving diets containing different additives^*^. *CON: basal diet formulated to meet the nutritional requirements of each phase, without the inclusion of antibiotic; ATB: basal diet with inclusion of antibiotic, specifically, lincomycin and colistin; FA: basal diet with inclusion of probiotic and multienzyme blend, the probiotic was *Bacillus subtilis* (3 × 10^8^ CFU/g of product) and a multienzyme blend of exoglycanase, endoglycanase, protease, pectinase, polygalacturonidase, and β-amylase (BioAmin – FATEC/Trouw Nutrition^®^); AB + FA: basal diet with inclusion of antibiotic, probiotic and multienzyme blend

No treatment effect on fecal score was observed during the first trial – Fig. 1. For the belly nosing behavior assessment, piglets on the AB + FA group had 20.17% less total belly nosing events (*p* = 0.025) compared to piglets in the CON group – Table 3.

**Table 3:** Total piglets presenting belly nosing behavior, observed from 7 am to 8 am and from 4 Pm to 5 Pm, receiving diets containing different types of additives

Event Frequency	1	2	3	4	5	6	7	8	Total events
Treatments	Number of pigs per frequency								
CON	10	10	10	20	16	10	11	13	461^a^
AB	16	19	09	0	16	19	22	0	429^ab^
FA	08	13	19	25	10	25	0	0	391^ab^
AB + FA	04	29	24	16	0	12	14	0	368^b^

Means followed by different superscript lowercase letters were significantly different in Dunn’s test (*P* = 0.025)

^†^CON: basal diet formulated to meet the nutritional requirements of each phase, without the inclusion of antibiotic; ATB: basal diet with inclusion of antibiotic, specifically, lincomycin and colistin; FA: basal diet with inclusion of probiotic and multienzyme blend, the probiotic was *Bacillus subtilis* (3 × 10^8^ CFU/g of product) and a multienzyme blend of exoglycanase, endoglycanase, protease, pectinase, polygalacturonidase, and β-amylase (BioAmin – FATEC/Trouw Nutrition^®^); AB + FA: basal diet with inclusion of antibiotic, probiotic and multienzyme blend

### Digestibility trial

In the digestibility trial, the only difference observed was on NABS – Table 4. Piglets from the FA group had 7.14% higher (*p* = 0.024) nitrogen absorption than piglets from the CON group.

**Table 4:** Dry matter digestibility coefficient (DRDC), gross energy digestibility coefficient (GEDC), digestible energy (DE), metabolizable energy (ME), retained nitrogen (NRET), absorbed nitrogen (NABS) and the relationship between retained and absorbed nitrogen (NRETABS) in pig diets in the initial phase containing different additives^†^

Variable	CON	AB	FA	AB + FA	SEM^*^	Cohen’s d	*P-Value*
	Energy balance						
DMDC (%)	80.55	79.44	81.25	81.95	1.778	-	0.200
EBDC (%)	83.08	80.24	82.92	81.94	2.625	-	0.064
DE kcal/kg	3147	3105	3164	3202	70.030	-	0.229
ME kcal/kg	3023	3018	3070	3107	84.304	-	0.334
	Nitrogen Balance						
NRET (%)	52.89	52.41	58.05	57.79	3.826	-	0.069
NABS (%) ^*^	73.11^b^	73.95^ab^	78.33^a^	77.34^ab^	2.672	0.708	0.024
NRETABS (%)	72.19	70.79	74.37	75.02	3.735	-	0.300

Means followed by different superscript lowercase letters were significantly different in by the Tukey test (*P* < 0.05)

^*^SEM = standard error of the mean

Effect sizes (Cohen’s d) were calculated for the pairwise comparison between the CON and AB + FA groups, reflecting the lowest and highest treatment responses, respectively

^†^CON: basal diet formulated to meet the nutritional requirements of each phase, without the inclusion of antibiotic; ATB: basal diet with inclusion of antibiotic, specifically, lincomycin and colistin; FA: basal diet with inclusion of probiotic and multienzyme blend, the probiotic was *Bacillus subtilis* (3 × 10^8^ CFU/g of product) and a multienzyme blend of exoglycanase, endoglycanase, protease, pectinase, polygalacturonidase, and β-amylase (BioAmin – FATEC/Trouw Nutrition^®^); AB + FA: basal diet with inclusion of antibiotic, probiotic and multienzyme blend

## Discussion

The nutritional transition after weaning decreases piglets’ feed intake and compromises intestinal health and function (Tang et al. 2022). Weaning down-regulates the expression of glycolysis and citrate cycle proteins in the enterocytes, which compromises energy metabolism, leads to cell death and immune activation (Cao et al. 2018; Yang et al. 2016). Combined with the stress from changes in housing and mixing (Van Kerschaver et al. 2023), this contributes to a pro-inflammatory state following weaning.

To alleviate these symptoms, antibiotics have been used to control bacterial growth and improve immune status throughout a pig’s life. Reports show that antibiotics can improve productive performance (Cao et al. 2019; Li et al. 2021), average daily gain (ADG), feed intake (ADFI) and feed to gain ratio (F: G) when piglets are facing a sanitary challenge (Luise et al. 2019). However, they can also induce dysbiosis and compromise growth when their inclusion is unnecessary, such as when piglets are healthy or not under stress. Wang et al. (2019) recently reported that antibiotics increased the relative abundance of harmful bacterial in the colonic microbiome, such as *Campylobacteraceae*, and lowered gene expression of vitamins, amino acids, and glycans metabolic pathways. In such cases, antibiotics may inadvertently lead to diarrhea and negatively impact piglets’ performance (Kim et al., 2021 A).

As an alternative, feed additives such as probiotics and exogenous enzymes can potentially alleviate these unwanted side effects (Mekonnen et al. 2020). Probiotics have been reported to be capable of modulating piglet’s microbiota increasing Firmicutes, a group of bacteria related to healthy stools (Deng et al. 2020); enzyme complexes can help nutrient digestibility, lowering available substrate for bacterial fermentation and intestinal inflammation (Zhang et al. 2020a, b). Ichim et al. (2018) tested the potential of a combination of a probiotic complex (*Lactobacillus* and *Bifidobacterium* variants) and a multienzyme complex (including amylase, xylanase, and invertase) to mitigate the adverse effects of cancer drugs and antibiotic in human intestinal models. The authors reported an improved colon fermentation profile, a restored Bacteriodetes/Firmicutes ratio in in vivo and reduced intestinal inflammation in vitro, showing the capacity of these feed additives to improve medication-induced dysbiosis and inflammation.

In pigs, the association of *Enterococcus faecium* and xylanase has been reported to improve ADG and FCR in growing pigs (Nguyen and Kim 2020). Under *E. coli* challenge, xylanase and probiotics reduced inflammatory cytokines in the intestines, increased villus height, and lowered diarrhea incidence (Duarte et al. 2020). The authors associated this effect to the release of carbohydrates by xylanase, which were previously unavailable for *B. subtilis* fermentation, enhancing the probiotic effect. Besides, *B. subtilis* can help induce intestinal maturation and increase villus height (Deng et al. 2020), which, together with the increased nutrient release promoted by the enzymes, increases absorption and promotes growth.

In a recent study comparing a multienzyme blend, to chlortetracycline and untreated pigs, Li et al. (2021) reported both the first two groups improving BW, ADG, and F: G. Han et al. (2017) also tested a similar enzymatic blend at various inclusion levels, with or without colistin and aureomycin, and found a positive impact on ADG when antibiotics were associated with enzymes. In both cases, performance results were followed by improved intestinal inflammatory status or increased endogenous enzymes activity. Our findings seem to agree with overall reports, since both feed additives alone (FA) and associated with antibiotics (AB + FA) improved performance in relation to untreated pigs. While the FA association yielded positive results, we cannot separate induvial feed additive effects due to our experimental design. As such, we cannot make any conclusion about the nature of said effect, e.g. additive or multiplicative, only speculate.

It is important to note, however, that contradictory effects have also been reported by other authors. Sudan et al. (2023) compared two diets supplemented with different concentration of *B. subtilis* to diets containing 3000 mg/kg of zinc oxide (ZnO) and found that the probiotic fed groups did not outperform any of the other treatments, including the unsupplemented negative control. Similarly, pigs supplemented with another *Bacillus* strain under two different timing regimens had worse productive performance than the antibiotic (apramycin) plus ZnO (2500 mg/kg) group, which was comparable to our AB group (Crespo-Piazuelo et al. 2021).

While both studies share similarities with ours, the additive concentrations varied widely, which may explain the differences in outcomes. The probiotic dose used in our trial was intermediate between those tested in the other studies, whereas our ZnO concentration was considerably lower. Moreover, unlike the referenced studies, we combined ZnO with probiotics. Nonetheless, other authors have also reported inconsistent findings when evaluating single or multi-enzyme supplementation, with treated groups performing similarly to untreated controls (Atoo et al. 2025; Munezero and Kim 2022; Peng et al. 2022), even when several other reports indicate that enzyme supplementation leads to improved nutrient digestibility and performance (Chen et al. 2020; Duarte et al. 2020; Long et al. 2021; Soderstrom et al. 2024; Zhao et al. 2020).

In the present study we observed improved nitrogen absorption in piglets fed diets supplemented with enzymes. Among the enzymes tested, proteases can directly increase nitrogen absorption by releasing amino acids previously undigested (Yu et al. 2020). Similarly, carbohydrases, digesting non starch polysaccharides, can also release associated proteins (Boontiam et al. 2022). Kim et al. (2021B) reported that the inclusion of 300 mg/kg of protease in the diet of growing pigs reduced nitrogen excretion in urine and feces, while nitrogen absorption tended to increase. Since we did not include single enzyme groups in our experiment, we cannot affirm which enzyme was responsible for the improved digestibility, or even if the effect is due to the association.

Other published results are not as consistent in reporting improved protein digestibility due to enzyme supplementation. Atoo et al. (2025) compared the performance and apparent ileal digestibility (AID) of amino acids of piglets fed with a low soybean meal diet (17% in the first phase) or a high soybean meal diet (30% in the first phase) supplemented with an enzymatic blend which included, among others, glucanase, protease, pectinase, and amylase. No differences in the AID of the main amino acids were reported, which also translated into similar performance among groups, even though piglets fed the high–soybean meal diet received higher crude protein levels. Munezero and Kim (2022) reported similar digestibility results when using only a protease in the feed.

Nonetheless, the observed increase in nitrogen absorption might have been the cause of the higher performance in the feed additive groups. Nitrogen is essential for protein accretion and enhanced absorption is often associated with improved performance (Kim et al. 2012). During weaning, protein accretion is compromised due to a pro inflammatory state, as a portion of dietary amino acids is redirected toward the synthesis of immune cells and cytokines (Klasing 2017; Obled 2003). Protein restriction, for example, jeopardizes acute phase immune response, showcasing the importance of nutritional protein supply in inflammatory responses (Hoek et al. 2015). Nutritional strategies aimed at modulating inflammation, may indirectly enhance growth by improving health status and optimizing protein utilization.

This remains a speculative mechanism in the present study, as we have not made any direct assessment of inflammatory markers. Besides, higher nitrogen absorption did not cause higher performance in the AB group, which exhibited the poorest productive performance among all treatments. Despite the lack of statistical evidence, the AB group presented the lowest NRET, which could suggest inefficient utilization of the absorbed protein for tissue deposition. Hoek et al. (2015) observed that, in immune-challenged pigs fed adequate levels of protein, inflammation reduced nitrogen retention by increasing urinary nitrogen excretion. While speculative, previous research has reported immunomodulatory benefits associated with similar treatments, supporting the plausibility of this mechanism (Galli et al. 2024; Sampath et al. 2024; Valente Junior et al. 2024).

Independently of the mechanism that impeded AB performance to improve even while absorbing more nitrogen, a decrease in performance of antibiotic fed piglets is uncommon. In the majority of published reports, antibiotic treatment alone is typically associated with superior performance, at least compared to untreated controls (Li et al. 2021; Silva et al. 2010). We believe that the inclusion of ZnO, which at higher doses (i.e., over 2500 ppm) can increase the evenness (Mezzina et al. 2025) and occurrence of antibiotic-resistant genes, may have reduce the overall trial sanitary challenge and consequently the effectiveness of antibiotics (Johanns et al. 2019; Vahjen et al. 2015).

Similar to our results, Lima Neto et al. (2018) reported that antibiotics initially worsened the performance of healthy, unchallenged finishing pigs. As the pigs continued to fatten, stress levels increased, and their sanitary status declined, leading to a subsequent improvement in performance with the inclusion of antibiotics. Since our facilities were kept clean throughout the trial and pen space was never insufficient, combined with the inclusion of ZnO, may have contributed to the unexpected effect of antibiotics. However, this hypothesis might not be the sole reason for this antibiotic related result, as the before-mentioned Crespo-Piazuelo et al. (2021) study reported antibiotics supplemented with ZnO outperforming probiotic supplementation without any sanitary challenge to the piglets. Thus, the result we are reporting here might be attributed to other, non-predicted variables, which were not evaluated with this trial’s experimental design.

The fecal score averages observed also indicate the low environmental challenge faced by the piglets, since all groups, including the untreated control group (CON), presented healthy stools during the whole trial. It is well reported that probiotics, enzymes, and antibiotics decrease fecal score and diarrhea incidence (Duarte et al. 2020; Tian et al. 2021), due to microbiota modulation and higher nutrient digestibility (Duarte et al. 2020; Mekonnen et al. 2020). Reports, however, also indicate that feed additives’ health promoting effects are more prominent when piglets face higher sanitary challenge (Upadhaya et al. 2019), with, in some cases, probiotic fed piglets displaying improved performance only in the weeks they were challenged (Guimarães et al. 2024). Post-weaning diarrhea is a common occurrence in the nursery phase (Eriksen et al. 2021) and the fact that even the CON group did not show high diarrhea frequency, is an indicator that there was not enough environmental challenge to observe the additives’ effects.

Although probiotics have demonstrated beneficial effects in modulating host responses under stressful conditions, such as infections with enterotoxigenic *Escherichia coli* (ETEC) in pigs (Roselli et al. 2017), their supplementation in nursery diets has produced inconsistent outcomes (Veizaj-Delia et al. 2010; Satessa et al. 2020). In a trial which similarly to ours tested a probiotic and enzymatic blend association, Chen et al. (2016) observed that the feed additives improved fecal score and performance only before *E. coli* challenge; after the inoculation, the combination equalized with the untreated challenged groups. Our results, together with previous findings, highlight the inconsistency of feed additive responses, even under controlled experimental conditions. Further research conducted in uncontrolled environment, closer to the condition’s piglets experience on commercial farms, is needed before any assertion can be made regarding their on-farm effects.

A possible reason for this inconsistency is the timing of administration: when probiotics are introduced shortly before weaning, there may be insufficient time for effective intestinal colonization, thereby reducing their potential to mitigate the dysbiosis typically triggered by this critical transition (Barba-Vidal et al. 2018). In neonatal piglets, the immature gastrointestinal tract represents a window of opportunity to guide the composition of the gut microbiota and intestinal maturation (Choudhury et al. 2021), both being highly sensitive to external factors such as nutrition, antimicrobial use, and environmental stressors (Schokker et al. 2014). During this period, the high plasticity of the microbial community allows for the strategic administration of beneficial probiotic strains, aiming to support the establishment of a balanced microbial ecosystem (Hashemi et al. 2016). Early microbial colonization of the intestine plays a crucial role in host physiology and development, contributing to the definition of its phenotype. Early intervention has the potential to enhance piglet resilience to weaning-related challenges by promoting a more stable intestinal environment, stimulating immune system development, and increasing the host’s ability to resist post-weaning infections (Hansen et al. 2022).

Belly nosing is a stereotypical, stress-related behavior frequently observed in recently weaned piglets (Dybkjær 1992) which can decrease weight gain of the animals performing it (Straw 2001). Few studies have evaluated if or how feeding patterns and diet composition can affect repetitive stress behaviors. A feed additive based on marine extract reduced the time spent executing stress associated behaviors, including belly nosing, and decreased salivary cortisol levels (O’Driscoll et al. 2013). Bruni et al. (2008) observed that restricting feed after a period of plentiful eating led to an increase in belly nosing behaviour. Belly nosing resembles the udder massage piglets perform to stimulate milk production in sows (Dybkjær 1992), hence, the reduction in available feed caused the heightened frequency of foraging-like behaviours.

Anorexia during the nursery diet transition creates a scenario of restriction similar to that described by Bruni et al. (2008), thus, feed additives that ease the nutritional adaptation and accelerate feeding reestablishment, could potentially lower belly nosing. This theory is strengthened by the fact that, in the case of Bruni et al. (2008), the increase in belly nosing was accompanied by a decrease in time spent eating. While in the present study, the combination of antibiotics, probiotics, and enzymes were associated with reduced belly nosing frequency, our behavioral ethogram was limited, and we cannot properly confirm how piglet time budget changed across groups. Therefore, we cannot assert that the apparent observed change was due to increased time spent feeding and this hypothesis remains speculative.

Time budget changes are also not the only possible explanation for the belly nosing decrease observed, since general health also influences the behavior frequency. Straw et al. (2001) reported that prostrated piglets are often targeted for belly nosing, and prostration is one of the primary behaviors observed in cases of illness (Paris-Garcia and Wagner 2021). Besides, another stress-associated behaviour, tail biting, has been linked to disease, indicating that health influences social interactions among pigs (Boyle et al. 2022). Several studies have reported that probiotics, enzymes and antibiotics can have a profound impact on intestinal and general health by preserving intestinal barrier, increasing villus height, lowering the concentration of inflammatory cytokines and pro inflammatory microbial metabolite production (Lee et al. 2020; Tiwari et al. 2018; Zhang et al. 2020a, b). Probiotics can also lower piglet susceptibility to illness, by decreasing intestinal *E. coli* colonization due to changes in intestinal receptors expression (Daudelin et al. 2011). Post weaning dysbiosis is associated with intestinal inflammation and enteric disease (Gresse et al. 2017), which can increase prostration (Veit et al. 2021) and make sick piglets more vulnerable to belly nosing.

Diet is also one of the main influencers of the microbiota-gut-brain axis, which could also be related to the results observed (Kobek-Kjeldager et al. 2022). In other species such as mice and humans, microbiota modulation can influence stress and anxiety response (Hsiao et al. 2013; Kraimi et al. 2019), and in pigs, has also been associated with tail biting. By improving intestinal and general health, feed additives could indirectly lower belly nosing by decreasing the number of piglets susceptible to this behavior.

This research topic and its connection to animal welfare is still in its early stages; it is important to emphasize that further studies on the influence of diet on behavior are needed. To the authors knowledge, no other trial evaluated the association of feed additives on belly nosing reported here. New studies have been researching the relationship between diet composition, especially amino acid levels (Kwon et al. 2022; Lay et al. 2021; Wang et al. 2023), with stress behaviors and our report is another indicative that there is merit in exploring other nutritional strategies to ease piglet stress during the post weaning nutritional transition. A more holistic approach is recommended, where nutritionists consider not only the impact of diets on productive performance but also their broader effects on animal behavior, health. and overall well-being.

Recently weaned piglets still have an immature gastrointestinal tract, thus digestive enzyme production is lower, and pH levels are not acidic enough for proper enzyme action (Hedemann and Jensen 2004). Low secretion of pancreatic enzymes such as trypsin, chymotrypsin, and amylases hinder digestion and absorption leading to the growth stagnation immediately after weaning, which is potentialized by the nutritional transition. While this challenge is more prominent during the first two weeks of nursery, growing and finishing pigs are still not capable of fully digesting their diet, therefore exogenous enzymes can still have a positive effect even in older pigs (Lee et al. 2019). Several authors have demonstrated enzymes’ capacity to increase digestibility of dry matter (DM), energy, crude protein (CP), ether extract (EE), and ash (Boontiam et al. 2022; Yu et al. 2020;). Kiarie et al. (2020) reported digestibility of DM, gross energy, CP and EE 3.6%, 5.1%, 3.5%, and 15.7% higher in pigs fed a multienzyme complex of xylanase, β-glucanase, protease, amylase and others.

While published data indicates that multienzyme supplementation can increase energy digestibility (Boontiam et al. 2022; Kiare et al. 2020), this effect was not observed in this study. Enzymes are substrate dependent (Ravindran 2013), thus their action is influenced by diet formulation. In our trial, we offered sources of highly digestible carbohydrates, such as gelatinized corn, bakery by products, dextrose and sugar, not added by the before cited authors in their formulations. While the diets practiced in this study follow commonly adopted practices for recently weaned piglets (Carnino et al. 2025), it is possible that their formulation influenced available substrate for enzyme action. Other reports indicated that including ingredients rich in the substrate targeted by the enzyme improved its effects in vivo (Boontian et al. 2022). The increase in protein absorption could also be explained by this hypothesis, since protein bioavailability in our diets was lower.

It is important to note that while the feed additives results presented in this and many other studies indicate their positive effect on pigs, some risk remains. Excessive use of enzymes can cause diarrhea due to increased osmotic concentration in the lumen (Moeser and Blikslager 2007). Protease is also an enzyme that needs further studying, since some reports indicate that in high doses it can also cause intestinal wall lesions (Amer et al. 2021). In a still not published study conducted by the present group, protease fed piglets displayed villus atrophy and increased expression of apoptosis proteins Fas and Caspase 8. Although a large body of research highlights the positive roles of probiotics including their capacity to enhance gastrointestinal health, modulate the gut microbiota, and inhibit pathogenic organisms, there is also a growing number of studies reporting neutral or adverse effects such as decreases in performance, associated with higher diarrheal frequency and drop in serum immunoglobulin levels (Trevisi et al. 2011) or increased pathogen translocation (Kreuzer et al. 2012).

In the controlled, experimental environment of the present study, the inclusion of probiotics and a multienzyme blend in the diet of weanling pigs appears to mitigate negative effects associated with weaning stress. While we have reported promising results, some key limitations of our study need to be mentioned, such as the small sample size we tested and the low environment sanitary challenge. Piglets face much more challenging conditions in commercial farms, and as such future studies should evaluate these feed additives on similar conditions to confirm their potential to replace antibiotics in commercial production systems. We also recommend the inclusion of single additive groups in the experimental design, as to better elucidate the nature of the effects caused by combinations. Additionally, investigating further immunological, inflammatory, microbiome among other variables will help the scientific community understand how these feed additives are modulating their effects on animals. A holistic approach remains essential for successful application in practice, considering the interaction among genetics, diet formulations, additive dosage, and environmental factors when designing nutritional strategies.

## Data Availability

The datasets generated during the current study are not publicly available but are available from the corresponding author on reasonable request.
